# Predicting non-surgical treatment failure in patients with spontaneous pneumothorax—the Base-C score: a retrospective study

**DOI:** 10.7717/peerj.21288

**Published:** 2026-06-18

**Authors:** Xiangli Shao, Lijing Yu, Zhaofeng Huang, Jie Weng, Lielie Zhu, Can Jin

**Affiliations:** 1 Emergency Medicine, The Second Affiliated Hospital and Yuying Children’s Hospital of Wenzhou Medical University, Wenzhou, China; 2 Pediatric Urology, The Second Affiliated Hospital and Yuying Children’s Hospital of Wenzhou Medical University, Wenzhou, China; 3 General Practice, The Second Affiliated Hospital and Yuying Children’s Hospital of Wenzhou Medical University, Wenzhou, China; 4 Rehabilitation, Wenzhou TCM Hospital of Zhejiang Chinese Medical University, Wenzhou, China

**Keywords:** Spontaneous pneumothorax, Non-surgical treatment failure, Predictive model, Base-C score, Risk stratification

## Abstract

**Background:**

The probability of failure for non-surgical treatment of spontaneous pneumothorax (SP) is relatively high. Early identification of patients at risk of non-surgical treatment failure, with timely intervention, can lead to improved outcomes. This study aimed to develop a model to identify patients at high risk of non-surgical treatment failure, thereby personalizing treatment strategies and improving patient outcomes.

**Methods:**

We conducted a retrospective cohort study using data from two large comprehensive hospitals. Demographic data, comorbidities, imaging findings and clinical outcomes were collected. The primary outcome was treatment failure, defined as the need for surgical intervention or the failure of non-surgical management to resolve the pneumothorax within 8 weeks. We employed multivariable logistic regression to identify risk factors and established the Base-C score based on the regression coefficients, which was validated externally.

**Results:**

In the present study, the derivation cohort consisted of 1,378 patients, and the validation cohort comprised 506 patients. The Base-C score included pulmonary bullae, age, smoking, emphysema and the degree of lung collapse. The Base-C score demonstrated excellent discriminative ability in predicting SP treatment failure, with an area under the curve (AUC) of 0.836 (95% confidence interval (CI) [0.813–0.860]) in the derivation cohort and 0.829 (95% CI [0.791–0.866]) in the validation cohort. The Hosmer-Lemeshow (H-L) goodness-of-fit test and calibration curves indicated good calibration in the derivation and validation cohorts. In the clinical application context, Decision Curve Analysis (DCA) demonstrates that the Base-C score generates a positive net benefit for outcomes when the threshold probability is greater than 15%.

**Conclusions:**

The Base-C score showed promise as an effective tool for identifying the risk of non-surgical treatment failure in patients with SP. It may aid in guiding clinical management strategies and has the potential to improve patient outcomes, though this requires prospective verification.

## Background

Spontaneous pneumothorax (SP) is a common pulmonary disorder characterized by the presence of air in the pleural space without apparent external injury (Hallifax et al., 2018; Noppen, 2010). The conventional management strategies for SP include observation with supplemental oxygen and closed pleural drainage, which are less invasive and associated with faster recovery times (Walker et al., 2024; Vuillard et al., 2019; Shaireen et al., 2014). However, a significant proportion of patients experience treatment failure, necessitating more aggressive interventions such as thoracoscopic surgery (Sawada, Watanabe & Moriyama, 2005; Tschopp et al., 2006). The ability to early identify patients who are likely to fail non-surgical management could facilitate timely surgical intervention, potentially reducing healthcare costs, shortening hospital stays, and, most importantly, improving patient outcomes.

Previous studies have attempted to identify predictive factors for the failure of non-surgical treatment in SP, such as a history of prior ipsilateral pneumothorax, a high degree of lung collapse, and the presence of radiological bulla formation (Nakano et al., 2023; MacDuff, Arnold & Harvey, 2010). A systematic review and meta-analysis by Walker et al. (2018) aimed to determine the recurrence rates in primary SP, which is crucial for understanding the prognosis and planning treatment strategies. Another study by Aguinagalde et al. (2018) presented clinical practice guidelines on the management of SP, which included an assessment of various factors influencing treatment outcomes. These studies underscore the importance of identifying patients who are at high risk of treatment failure to facilitate timely surgical intervention. However, there are few reports about predictive models for predicting the failure of non-surgical treatment of pneumothorax.

By identifying patients who are unlikely to benefit from non-surgical treatment management, clinicians can escalate treatment plans more promptly. Furthermore, failure of non-surgical treatment can lead to prolonged hospitalization and the need for secondary interventions, which not only increases the organizational burden and financial costs on the healthcare system but also causes additional pain and inconvenience to patients. Therefore, early and accurate identification of high-risk patients is crucial for optimizing the allocation of medical resources, reducing socioeconomic burdens, and improving patient experience. The objective of this study is to develop and validate a predictive model for the failure of non-surgical treatment in patients with SP. This model aims to integrate clinical and radiographic to identify patients at high risk of treatment failure, thereby personalizing treatment strategies and improving patient outcomes.

## Methods

### Study design and participants

We conducted a retrospective cohort study utilizing data from two large comprehensive hospitals. The study period for the derivation cohort was from June 1, 2014, to May 31, 2024. The validation cohort data were collected from June 1, 2017, to May 31, 2024. This study included patients aged ≥18 years who were diagnosed with SP and initially received non-surgical treatment. Non-surgical treatment was explicitly defined as one or more of the following modalities: observation with supplemental oxygen, needle aspiration, or closed thoracic drainage. Patients with the following criteria were excluded: those who directly received surgical treatment such as video-assisted thoracoscopic surgery (VATS) upon admission, did not undergo imaging review, those with trauma-related pneumothorax and those with incomplete data on their first imaging characteristics.

### Patient identification

Eligible patients were identified through a search of the electronic medical records (EMR) system at both participating centers, utilizing International Classification of Diseases (ICD) codes related to SP and keywords associated with pneumothorax. The EMRs of potential participants were independently reviewed by two researchers to ensure that they met the established inclusion and exclusion criteria. This study was conducted in accordance with the ethical standards of the responsible committees on human experimentation and with the Helsinki Declaration of 1975. This study is retrospective in nature, therefore informed consent was waived. This study was approved by the Ethics Committee of the Second Affiliated Hospital and Yuying Children’s Hospital of Wenzhou Medical University (Ethical Application Ref: IRB# 2024-K-148-01).

### Sample size

The sample size for the study was determined based on the Events Per Variable (EPV) principle, which recommends at least 10 events per predictor variable for logistic regression modeling (Vittinghoff & McCulloch, 2007). Given that the non-surgical treatment failure rate for SP is approximately 35%, we estimated that a total of 500 patients in the modeling cohort would provide about 175 cases of treatment failure. For the validation cohort, we aimed to include at least 200 patients to ensure that the model’s performance could be externally validated and generalized to an independent cohort.

### Variables

A comprehensive set of clinical, demographic, and imaging variables was considered for inclusion in the predictive model. The candidate variables were chosen based on their clinical relevance, supported by prior studies and expert opinions. These variables included: Demographic data: age, gender, body mass index (BMI), family history of pneumothorax, and smoking history. Comorbidities, including chronic lung diseases such as chronic obstructive pulmonary disease (COPD), emphysema, pulmonary fibrosis, and past history of tuberculosis. Pneumothorax characteristics: the degree of lung collapse (classified according to the Japan Society for Pneumothorax and Cystic Lung Disease guidelines (Nakano et al., 2023)), pneumothorax location, and presence of large bullae or other lung lesions on imaging. Imaging features: the presence of lung bullae, pulmonary nodules, or any other abnormal findings on chest X-ray and CT scan.

Lung collapse was categorized as small, moderate, or large based on the size of the pneumothorax and the extent of lung collapse (Nakano et al., 2023). The presence of lung bullae was determined from chest CT imaging, and pulmonary nodules were assessed based on the findings from imaging studies. For imaging analysis, the pneumothorax size was measured using the largest diameter and classified as small (<3 cm), moderate (3–5 cm), or large (>5 cm).

To ensure consistency and reliability in imaging interpretation, all chest CT scans were independently reviewed by two experienced thoracic radiologists who were blinded to the clinical outcomes. A standardized data collection form was used to document the findings. Any discrepancies between the initial assessments were resolved through consensus or, if necessary, by adjudication from a third senior radiologist.

### Outcome

The primary outcome was non-surgical treatment failure, defined as: (1) the need for surgical intervention (*e.g*., video-assisted thoracoscopic surgery or pleurodesis), or (2) failure of non-surgical management to resolve the pneumothorax within 8 weeks (Walker et al., 2024; Ennis & Dobler, 2020). Failure was determined by a composite endpoint including: (1) follow-up chest imaging (X-ray or CT) at 8 weeks showing persistent pneumothorax (lung collapse greater than a small degree); (2) persistent clinical symptoms (*e.g*., chest pain, dyspnea), and/or (3) clinician judgment that further invasive intervention was required. Non-surgical treatment was defined as observation with supplemental oxygen and/or closed pleural drainage, without surgical intervention.

### Statistical analysis

Descriptive statistics were used to summarize continuous variables, which were presented as mean ± standard deviation (SD) or median with interquartile range (IQR), depending on their distribution. The differences between the derivation and validation cohorts were assessed using the Student’s t-test or Mann-Whitney U test for continuous variables and the chi-square or Fisher’s exact test for categorical variables.

To derive the predictive model for non-surgical treatment failure, logistic regression analysis was performed, with treatment failure (yes/no) as the dependent variable. Univariate logistic regression analyses were conducted to identify the unadjusted association between potential predictors and SP treatment failure (*P* < 0.05). For the SP treatment failure model, we used the backward stepdown logistic regression based on the smallest Akaike Information Criterion (AIC) value to identify the independent risk factors for SP patients’ treatment failure (Harrell, Lee & Mark, 1996). Prior to regression, multicollinearity among all candidate variables was assessed using the Variance Inflation Factor (VIF), with a VIF of <5 indicating no substantial collinearity. Variables were excluded from the final model primarily due to a lack of independent statistical significance (*P* ≥ 0.05) in the multivariate model, as determined by the stepdown procedure. Continuous variables were transformed into categorical variables based on clinical practice, and multiple logistic regression was performed to determine the final variables included in the scoring system. A scoring system was developed based on the final model by assigning a score to each variable according to its regression coefficient. The coefficient of each variable was divided by the smallest coefficient, and a corresponding score was assigned, with whole or half-point increments. Based on this, a nomogram prediction model was constructed.

The performance of the model was evaluated using the Area Under the Receiver Operating Characteristic Curve (AUROC). Model calibration was assessed with calibration curves and the Hosmer-Lemeshow (H–L) test and Brier score. DCA was used to evaluate the clinical application value of the model.

In addition to risk stratification, the optimal cut-off value for the Base-C score to predict treatment failure was determined by maximizing the Youden’s index (J = Sensitivity + Specificity −1) from the receiver operating characteristic (ROC) curve of the derivation cohort. The sensitivity, specificity, positive predictive value (PPV), and negative predictive value (NPV) corresponding to this optimal cut-off were calculated.

Missing data occurred primarily in some clinical variables. The missing rate for each variable was less than 5%. We used multiple imputation by chained equations (MICE) to handle missing data, assuming data were missing at random (MAR). The imputation model included all variables used in the analysis, and we conducted five imputations.

To ensure the broad applicability of the model, we performed pre-specified subgroup analyses to evaluate its performance separately in patients with primary spontaneous pneumothorax (PSP) and secondary spontaneous pneumothorax (SSP). The model’s discrimination, calibration, and clinical utility were assessed within each subgroup using the same methods as for the overall cohort.

All statistical analyses were conducted using R software (version 4.4.0; R Foundation for Statistical Computing, Vienna, Austria), with a significance threshold set at *P* < 0.05.

## Results

### Populations

**Figure 1:**
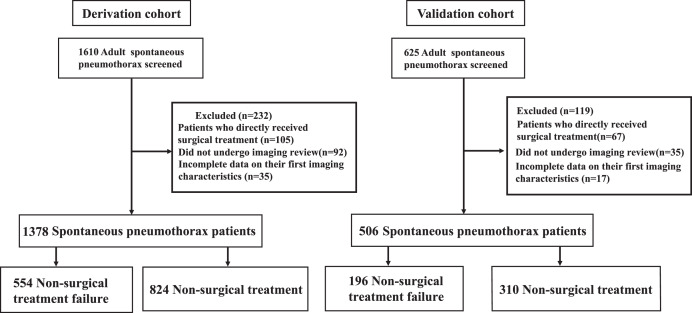
The study flow diagram.

The derivation cohort consisted of 1,378 patients, and the validation cohort comprised 506 patients (Fig. 1). In the derivation cohort, the incidence of treatment failure was 40.2%, while in the validation cohort, it was 38.7%, with no significant difference in the incidence of treatment failure between the two cohorts. This high failure rate, exceeding one-third of the patients, underscores the clinical challenge and the necessity for a reliable predictive tool. However, there were differences in baseline characteristics such as BMI, age, gender and smoking history (Table 1).

**Table 1:** Demographic and clinical characteristics of the patients with SP.

Variables	Derivation (*N* = 1,378)	Validation (*N* = 506)	*P*-value
**Demographics**			
Age (years), Median (IQR)	48 (36.0;63)	45 (37;59)	0.003
Male sex, *n* (%)	694 (50.4%)	307 (60.7%)	<0.001
BMI (kg/m^2^), Median (IQR)	25.3 (22.3;28.4)	24.0 (21.2;27.6)	<0.001
Family history of pneumothorax, *n* (%)	125 (9.07%)	53 (10.5%)	0.404
Smoking history, *n* (%)			0.024
No smoking	477 (34.6%)	186 (36.8%)	
Smoking time ≤ 10 years	329 (23.9%)	91 (18.0%)	
Smoking time > 10 years	572 (41.5%)	229 (45.3%)	
**Comorbidities**			
COPD, *n* (%)	97 (7.04%)	42 (8.30%)	0.407
Emphysema, *n* (%)	318 (23.1%)	82 (16.2%)	0.002
Pulmonary fibrosis, *n* (%)	66 (4.79%)	33 (6.52%)	0.169
History of tuberculosis, *n* (%)	67 (4.86%)	29 (5.73%)	0.521
**Imaging findings**			
Pneumothorax location, *n* (%)			0.671
Left-sided	687 (49.9%)	246 (48.6%)	
Right-sided	691 (50.1%)	260 (51.4%)	
Pulmonary bullae, *n* (%)	429 (31.1%)	167 (33.0%)	0.472
The degree of lung collapse, *n* (%)			0.009
Small	555 (40.3%)	181 (35.8%)	
Moderate	461 (33.5%)	156 (30.8%)	
Large	362 (26.3%)	169 (33.4%)	
Pleural effusion, *n* (%)	138 (10.0%)	52 (10.3%)	0.935
Pulmonary nodule, *n* (%)	137 (9.94%)	43 (8.50%)	0.392
Outcome			
Treatment failure, *n* (%)	554 (40.2%)	196 (38.7%)	0.600

**Note:**

BMI, Body Mass Index; COPD, Chronic Obstructive Pulmonary Disease.

### Predictors of SP non-surgical treatment failure

Univariate logistic regression was performed to identify potential risk factors for treatment failure in patients with SP. Factors such as age, smoking, pulmonary bullae, emphysema and the degree of lung collapse were found to be associated with treatment failure in SP patients. In the multivariate logistic regression analysis, these variables were identified as risk factors for treatment failure in patients with SP (Table 2).

**Table 2:** Logistic regression analysis results for SP patients treatment failure.

	Univariate logistic regression analysis			Multivariate logistic regression analysis		
Variable	β coefficient	Adjusted OR (95% CI)	*P*-value	β coefficient	Adjusted OR (95% CI)	*P*-value
Age	0.057	1.059 [1.051–1.067]	<0.001	0.023	1.023 [1.012–1.035]	<0.001
Male sex	−0.084	0.919 [0.741–1.140]	0.441			
BMI	−0.014	0.986 [0.962–1.010]	0.245			
Family pneumothorax history	−0.046	0.954 [0.652–1.386]	0.810			
Smoking history						
No smoking	Reference					
Smoking time ≤ 10 years	−0.193	0.825 [0.570–1.184]	0.301	0.123	1.131 [0.737–1.730]	0.572
Smoking time > 10 years	2.261	9.589 [7.219–12.838]	<0.001	1.579	4.852 [3.400–6.977]	<0.001
COPD	0.353	1.423 [0.923–2.190]	0.108			
Emphysema	2.853	17.347 [12.391–24.806]	<0.001	1.379	3.969 [2.445–6.523]	<0.001
Pulmonary fibrosis	0.171	0.843 [0.498–1.397]	0.515			
History of tuberculosis	0.386	1.472 [0.898–2.410]	0.123			
Pneumothorax location	−0.046	0.955 [0.770–1.184]	0.676			
Pulmonary bullae	2.698	14.851 [11.223–19.841]	<0.001	2.036	7.659 [5.348–11.074]	<0.001
The degree of lung collapse						
Small	Reference					
Moderate	1.118	3.060 [2.333–4.027]	<0.001	1.344	3.836 [2.653–5.602]	<0.001
Large	1.761	5.816 [4.351–7.816]	<0.001	1.832	6.249 [4.134–9.552]	<0.001
Pleural effusion	0.017	1.018 [0.708–1.452]	0.924			
Pulmonary nodule	0.064	1.067 [0.743–1.523]	0.724			

**Notes:**

Univariate and multivariate logistic regression analyses were performed to identify factors associated with treatment failure. Variables with a *P*-value < 0.1 in the univariate analysis were included in the multivariate model. Statistical significance was set at a two-sided *P*-value < 0.05. Blank cells in the multivariate analysis columns indicate that the variable was not retained in the final multivariate model.

### Base-C score and nomogram

**Figure 2:**
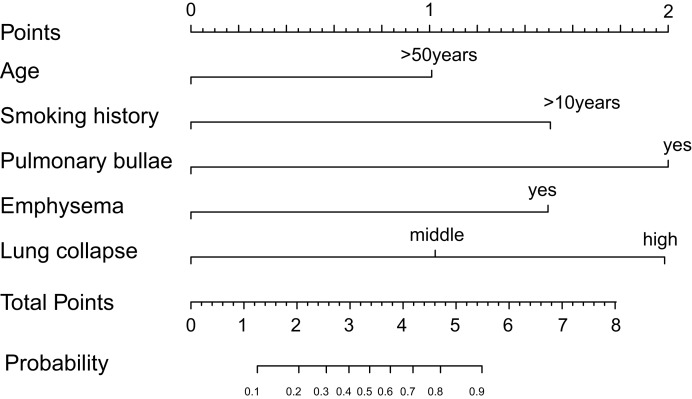
Nomogram for predicting SP treatment failure in the derivation cohort.

Based on our retrospective data, we have developed a novel scoring system for predicting treatment failure in patients with SP. We have named this scoring system the Base-C score, which encompasses five variables: pulmonary **B**ullae, **A**ge >50 years, **S**moking history >10 years, **E**mphysema and the degree of lung **C**ollapse (Table 3). The regression coefficients obtained from the multivariable logistic regression were used to calculate the score for each variable. The score for an individual patient was computed by summing the scores of each predictive variable applied to that patient. This cumulative score quantitatively assesses the risk of the patient experiencing treatment failure of SP. The total score ranges from 0 to 8, with higher scores indicating a greater risk of treatment failure for SP. To enhance clinical practicability and provide a more accurate description of the incidence of outcome events, we have constructed this scoring system into a nomogram prediction model (Fig. 2).

**Table 3:** Risk factors for predictive model for SP treatment failure in the derivation cohort.

Variable	β coefficient	Adjusted OR (95% CI)	*P*-value	Point^a^
Age				
>50years	0.934	2.543 [1.812–3.574]	<0.001	1
Smoking history				
Smoking time > 10 years	1.628	5.092 [3.573–7.318]	<0.001	1.5
Pulmonary bullae	2.075	7.966 [5.545–11.558]	<0.001	2
Emphysema	1.374	3.953 [2.496–6.339]	<0.001	1.5
The degree of lung collapse				
Middle	1.329	3.779 [2.607–5.532]	<0.001	1
High	1.873	6.511 [4.293–9.994]	<0.001	2
Total score				8

**Note:**

a Assignment of points to risk factors was based on a linear transformation of the corresponding β regression coefficient. The coefficient of each variable was divided by 0.934 (the smallest absolute β value, corresponding to Age) and allocated an integer or an half integer score for each variable.

### Risk stratification

Using the Base-C score for risk stratification of SP patients, the score is calculated by summing the scores of various risk factors. Risk stratification is divided into low-risk (0–3 points), moderate risk (3.5–5 points), and high-risk (5–8 points) groups. The selection of these specific cut-off points was data-driven, guided by the distribution of scores and event rates in the derivation cohort. The thresholds were chosen to approximate the quartiles of the score distribution among patients who experienced treatment failure, aiming to create risk groups with balanced sample sizes and clinically meaningful differences in outcome rates. In both the derivation and validation cohorts, the Base-C score effectively stratified patients into low-risk group, moderate-risk group, and high-risk group for SP treatment failure. The predicted incidence rates for each risk group closely matched the actual incidence rates (Table 4). To further validate the chosen cut-offs, we analyzed the observed failure rates across score deciles (Table S1). This analysis demonstrated a near-monotonic increase in risk, confirming that the thresholds of 3.5 and 5 points represent natural breakpoints that differentiate patients with low, intermediate, and high risk of non-surgical treatment failure.

**Table 4:** Risk of SP treatment failure in the derivation and validation cohort according to risk stratification.

Risk stratification	*n* (%)	Predicted risk (%)	Actual risk (%)
**Derivation cohort**			
Low	786 (57.04)	16.58 (15.33–17.84)	16.15
Moderate	278 (20.17)	49.23 (46.66–51.80)	51.07
High	314 (22.79)	91.32 (90.26–92.40)	90.76
**Validation cohort**			
Low	278 (54.94)	14.80 (13.07–16.53)	15.46
Moderate	166 (32.80)	60.71 (56.62–64.79)	58.43
High	62 (12.25)	87.23 (84.96–89.50)	90.32

**Note:**

The prognostic index was categorized in three groups: low-risk (0–3 points), moderate risk (3.5–5 points), high-risk (5–8 points).

### Validation of the Base-C score

**Figure 3:**
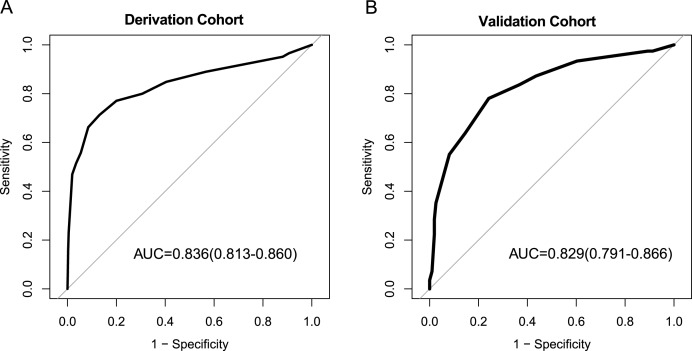
ROC curves for treatment failure prediction in derivation (A) and validation (B) cohorts.

**Figure 4:**
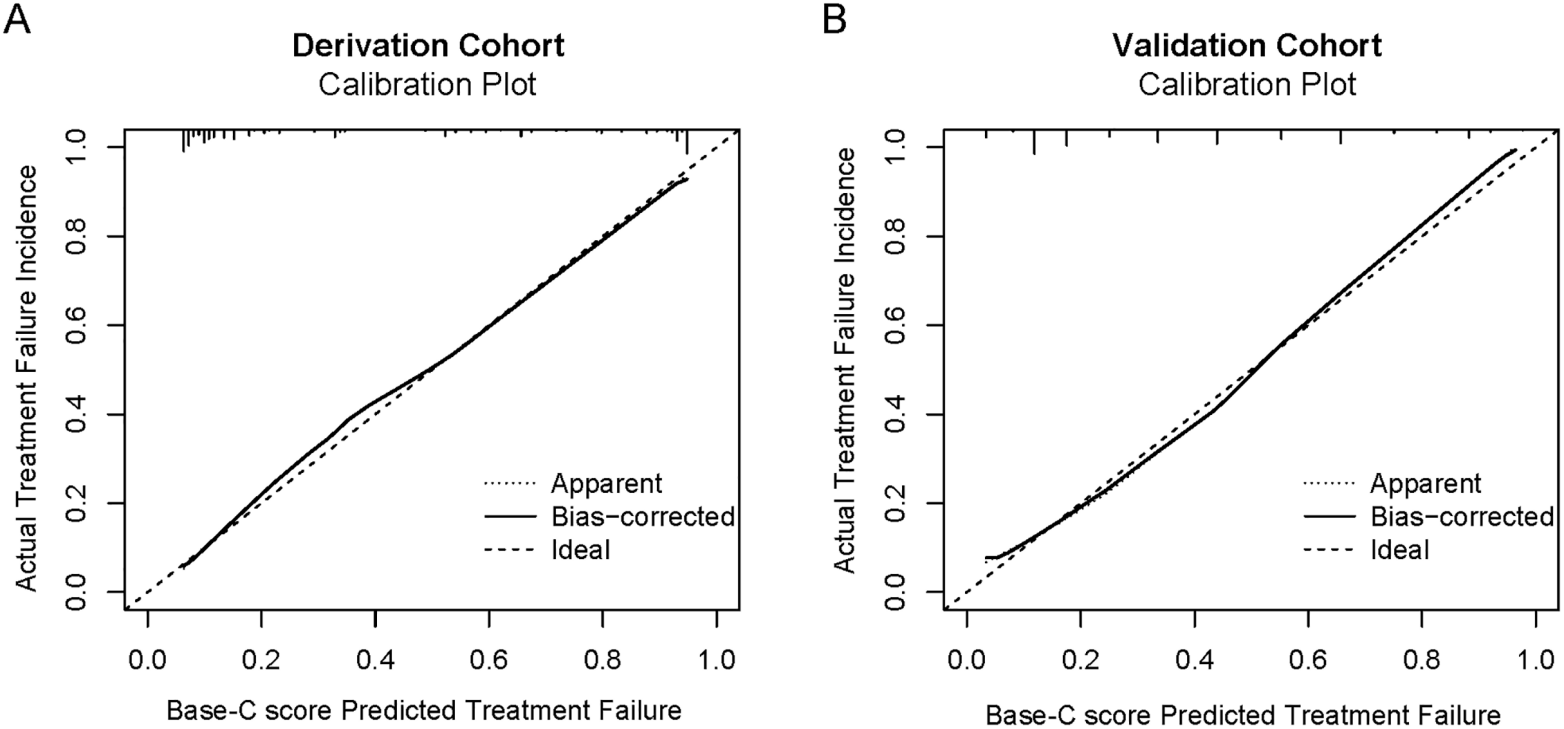
Calibration for treatment failure prediction in derivation (A) and validation (B) cohorts.

In the derivation cohort, the AUROC for the Base-C score was 0.836 (95% CI [0.813–0.860]) (Fig. 3A). In the validation cohort, the AUROC values for the Base-C score remained high at 0.829 (95% CI [0.791–0.866]) (Fig. 3B). We also assessed the calibration of the Base-C score through the Hosmer-Lemeshow (H–L) test and calibration curves. In the derivation and validation cohort, the Base-C score both demonstrated good calibration (derivation cohort: H–L chi-square value of 5.513, *P*-value of 0.597; validation cohort: H–L chi-square value of 2.572, *P*-value of 0.462). In the derivation and validation groups, the Brier scores of the prediction models were 0.1175 and 0.1214, respectively. The bias-corrected calibration curve generated by the bootstrap method showed a close fit to the reference line, indicating a high degree of consistency between the Base-C score’s predicted probabilities of SP treatment failure and the actual probabilities (Fig. 4).

To facilitate binary clinical decision-making, the optimal cut-off value for the Base-C score was identified as 4.5 points using the Youden’s index in the derivation cohort. At this threshold, the score demonstrated a sensitivity of 76.3% and a specificity of 76.8% for predicting treatment failure in the derivation cohort. The performance of this cut-off remained robust in the validation cohort, with a sensitivity of 74.6% and a specificity of 75.9% (Table S2).

### Net benefit of using the Base-C score

**Figure 5:**
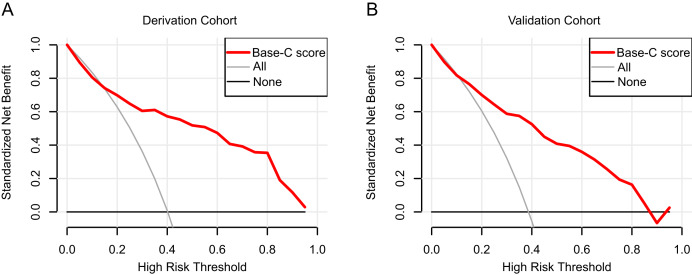
The DCA curve of treatment failure prediction in derivation (A) and validation (B) cohorts.

The Decision Curve Analysis (DCA) demonstrated the clinical utility of the Base-C score. As shown in Fig. 5, the model provided a positive net benefit over a clinically relevant range of threshold probabilities (approximately 15% to 90% in the derivation cohort). This indicates that if a clinician’s threshold for considering early surgical intervention falls within this range—meaning they would consider surgery for a patient with a predicted failure risk of, for instance, 20% or higher—using the Base-C score to guide decisions yields better clinical outcomes than a strategy of intervening in all or no patients (Table 5).

**Table 5:** Comparative net benefit of Base-C score in predicting treatment failure probability in SP.

	Derivation cohort			Validation cohort		
Threshold probability	Net benefit		Advantage of using Base-C score	Net benefit		Advantage of using Base-C score
	Treat all	Base-C score	Difference in net benefit	Treat all	Base-C score	Difference in net benefit
0	1	1	0	1	1	0
0.05	0.922	0.895	−0.027	0.917	0.898	−0.019
0.1	0.835	0.806	−0.029	0.824	0.817	−0.007
0.15	0.738	0.741	0.003	0.721	0.765	0.044
0.2	0.628	0.698	0.070	0.605	0.700	0.095
0.25	0.504	0.648	0.144	0.473	0.643	0.170
0.3	0.363	0.605	0.242	0.322	0.587	0.265
0.35	0.199	0.610	0.411	0.148	0.575	0.427
0.4	0.008	0.572	0.564	−0.054	0.526	0.58
0.45	−0.217	0.553	0.770	−0.294	0.450	0.744
0.5	−0.487	0.518	1.005	−0.582	0.408	0.990
0.55	−0.818	0.508	1.326	−0.933	0.395	1.328
0.6	−1.231	0.473	1.704	−1.372	0.360	1.732
0.65	−1.762	0.407	2.169	−1.937	0.314	2.251
0.7	−2.471	0.392	2.863	−2.690	0.257	2.947
0.75	−3.462	0.357	3.819	−3.745	0.194	3.939
0.8	−4.949	0.354	5.303	−5.327	0.163	5.490
0.85	−7.428	0.190	7.618	−7.693	0.051	7.744
0.9	−12.386	0.117	12.503	−13.235	−0.066	13.169
0.95	−27.26	0.029	27.289	−29.051	0.026	29.077

**Notes:**

This table presents the results of decision curve analysis (DCA) for the Base-C score model. The net benefit is calculated across a range of threshold probabilities, which represents the minimum probability of treatment failure at which a clinician or patient would opt for intervention. A higher net benefit indicates a better clinical utility. The “Difference in net benefit” shows the advantage of using the Base-C score model over the “Treat all” strategy. A positive value indicates that the model provides a superior net benefit at that threshold probability.

### Subgroup analysis

The Base-C score demonstrated consistent and robust performance across both PSP and SSP subgroups. Discrimination was good in PSP patients (AUC = 0.750 (95% CI [0.717–0.782])) and excellent in SSP patients (AUC = 0.889 (95% CI [0.862–0.916])) (Fig. S1). Calibration plots showed close to the ideal 45° line in both subgroups, with bias-corrected curves showing minimal optimism (Fig. S2). Decision curve analysis confirmed clinically meaningful net benefit for the Base-C score compared with “treat-all” or “treat-none” strategies across a wide range of reasonable threshold probabilities in both PSP and SSP patients (Fig. S3). These findings indicate that the score is not materially biased toward either subtype and can be applied reliably across the entire spectrum of spontaneous pneumothorax.

## Discussion

In this study, we observed that approximately 40% of patients with SP initially managed non-surgically experienced treatment failure. This substantial failure rate highlights a significant gap in current management strategies and confirms the urgent need for a tool to identify high-risk patients at the outset. The management of SP, particularly the selection between initial non-surgical and surgical strategies, remains a subject of ongoing refinement. To our knowledge, the Base-C score is the first validated predictive model designed specifically for this purpose, addressing a critical gap by integrating five routinely available predictors into a simple, clinically applicable tool.

Several studies have investigated predictors of non-surgical treatment failure. For example, Nakano et al. (2023) identified previous ipsilateral pneumothorax, large pneumothorax size (high degree of lung collapse), and visible bullae as independent predictors of initial treatment failure in a large retrospective cohort. Similarly, multiple observational studies and guidelines (including the 2010 British Thoracic Society guideline) have consistently associated larger pneumothorax size, bullous disease, and persistent air leak with higher rates of non-surgical failure (MacDuff, Arnold & Harvey, 2010). Meta-analyses and cohort studies have also linked smoking and advanced age to poorer response to non-surgical approaches, likely due to reduced parenchymal elasticity and increased structural vulnerability (Walker et al., 2018; Beghé et al., 2021; Nagata et al., 2020; Riveiro-Blanco et al., 2022; Bintcliffe et al., 2016). Landmark trials, such as the randomized conservative *versus* interventional management study by Brown et al. (2020) demonstrated that conservative management can be effective and non-inferior in selected patients with large primary spontaneous pneumothorax, but failure rates remain substantial (20–40% depending on definition and population), particularly in patients with unfavorable radiographic or clinical features. Recent reviews (2018–2024) confirm variable failure rates with non-surgical management and highlight the ongoing challenge of persistent air leak in both PSP and SSP, underscoring the need for personalized risk stratification tools to guide timely escalation. The selection of parameters for the Base-C score is firmly grounded in their pathophysiological roles. Advanced age and prolonged smoking history are associated with decreased lung compliance and increased parenchymal fragility (Beghé et al., 2021; Nagata et al., 2020), while bullae and emphysema directly impair lung re-expansion and promote persistent air leaks (MacDuff, Arnold & Harvey, 2010; Walker et al., 2018; Riveiro-Blanco et al., 2022; Bintcliffe et al., 2016). A large degree of lung collapse further challenges re-expansion efforts. Thus, the score likely captures a common pathway of impaired lung tissue integrity and reduced healing capacity that leads to non-surgical failure, transcending the traditional primary *vs*. secondary SP etiology. The robust performance of the Base-C score across both PSP and SSP subgroups enhances its generalizability.

The recent 2024 ERS/EACTS/ESTS clinical practice guidelines provide a comprehensive framework for managing spontaneous pneumothorax, emphasizing initial patient stratification based on symptoms and pneumothorax size to guide treatment selection (observation, aspiration, or drainage) (Walker et al., 2024). Our Base-C score directly complements this framework by offering a standardized, evidence-based tool for the critical next step: predicting which patients initially selected for non-surgical management are at high risk of failure. The Decision Curve Analysis confirmed the model’s clinical utility, showing a positive net benefit across a wide range of threshold probabilities (15–90%), indicating its value in guiding decisions for early surgical consultation, especially when clinicians face uncertainty.

The Base-C score has potential implications for clinical practice. By integrating easily obtainable demographic and imaging data, it enables efficient risk assessment in diverse healthcare settings. Furthermore, from an organizational and financial perspective, the accurate stratification of patients can prevent prolonged and ineffective non-surgical treatments, thereby reducing hospital stay durations and associated healthcare costs. Early surgical intervention for high-risk patients may ultimately be more cost-effective by avoiding complications from delayed treatment, such as hospital-acquired infections or prolonged disability. From a patient-centered viewpoint, avoiding a prolonged period of unsuccessful conservative management can significantly reduce physical discomfort, anxiety, and improve overall quality of life by facilitating a faster and more definitive recovery. The Base-C score leverages readily available data, making it particularly valuable in resource-limited settings. It can be integrated into electronic health records (EHRs) as a decision-support tool. Relevant patient data would automatically populate the scoring system, allowing clinicians to quickly assess treatment failure risk during routine care.

Despite the robust internal validation and promising performance of the Base-C score, our study has several important limitations. Foremost among these is its retrospective and two-center design. Although we identified strong and statistically significant associations between the predictors and the outcome, this design inherently prevents the establishment of causality. The relationships we observed should be interpreted as powerful predictive associations rather than definitive causal links. Therefore, we explicitly emphasize that the Base-C score is a predictive tool, and its components are risk indicators, not proven causative agents. Our study cohort encompassed patients receiving the full spectrum of non-surgical management (from observation to drainage). While this enhances the generalizability of our findings to the real-world clinical scenario of initial management selection, it introduces heterogeneity. However, this reflects the pragmatic reality of clinical practice, where treatment choice is stratified by severity. Our model aims to predict failure of the overall non-surgical pathway, and its robust performance across this heterogeneity suggests that the Base-C score captures fundamental risk factors for failure. Furthermore, the retrospective design carries an inherent risk of treatment-use bias (confounding by indication). It is possible that the clinical decision to proceed with early surgery was influenced by factors overlapping with the Base-C score predictors (*e.g*., large bullae), thereby creating a potential circularity where the predictors of ‘failure’ may have also prompted the intervention that defined the outcome. While this may inflate the model’s apparent accuracy, it also reflects the clinical reality the score aims to capture: identifying patients in whom clinicians intuitively anticipate non-surgical failure. As both participating centers are located within the same geographical region, the external validation may not fully capture the spectrum of SP etiology and management patterns seen in other populations (*e.g*., in Western countries). This geographic limitation highlights the need for future validation in broader, multi-regional cohorts. The relatively small size of the validation cohort might be underpowered to fully assess the Base-C score’s performance. This limitation could affect the model’s generalizability to broader populations. Additionally, unmeasured variables such as socioeconomic status, environmental exposures, genetic predispositions, and the presence of specific biomarkers were not included in our analysis but could further enhance predictive accuracy. Furthermore, while the SSP subgroup was of adequate size for model validation, its sample size may be suboptimal for developing a *de novo*, SSP-specific prediction model. However, the absence of significant interaction effects between pneumothorax type and the model’s predictors, coupled with its excellent validation performance in both subgroups, strongly supports the current strategy of employing a unified score. Our study acknowledges an important nuance in clinical application. The Base-C score identifies patients at high risk for non-surgical treatment failure, who may also have elevated operative risks due to underlying comorbidities such as advanced age and emphysema. Therefore, the score should not be interpreted as a direct indicator for surgery but as a tool to flag patients requiring more nuanced, individualized decision-making. The ultimate choice between pursuing a potentially prolonged non-surgical course or accepting the risks of surgery must involve a multidisciplinary evaluation of the patient’s overall condition, preferences, and local expertise. Future multicenter prospective studies are essential to validate the Base-C score in broader patient populations and explore its integration with emerging diagnostic technologies, such as quantitative lung imaging or artificial intelligence.

## Conclusion

In conclusion, this retrospective study suggests that the Base-C score represents a significant step forward in the management of SP. Its robust predictive capacity in our cohorts, combined with its ease of use, suggests it could become a valuable tool for guiding treatment strategies. If prospectively validated, enabling early identification of patients at risk of non-surgical treatment failure with the Base-C score could potentially improve outcomes, optimize resource use, and support personalized care for SP patients. However, its definitive impact on clinical practice awaits confirmation from future randomized controlled trials.

## Supplemental Information

10.7717/peerj.21288/supp-1 Supplemental Information 1 Calibration of the Base-C Score Across Risk Deciles in the Derivation Cohort.

10.7717/peerj.21288/supp-2 Supplemental Information 2 Predictive Performance of the Base-C Score at the Optimal Cut-off Value (≥4 points) Determined by Youden ’ s Index.

10.7717/peerj.21288/supp-3 Supplemental Information 3 The ROC curves of treatment failure prediction by the Base-C score in primary spontaneous pneumothorax (PSP) (A) and secondary spontaneous pneumothorax (SSP) (B) subgroups.

10.7717/peerj.21288/supp-4 Supplemental Information 4 The calibration curves of treatment failure prediction by the Base-C score in primary spontaneous pneumothorax (PSP) (A) and secondary spontaneous pneumothorax (SSP) (B) subgroups.

10.7717/peerj.21288/supp-5 Supplemental Information 5 The decision curve analysis (DCA) of treatment failure prediction by the Base-C score in primary spontaneous pneumothorax (PSP) (A) and secondary spontaneous pneumothorax (SSP) (B) subgroups.

10.7717/peerj.21288/supp-6 Supplemental Information 6 Raw data.

10.7717/peerj.21288/supp-7 Supplemental Information 7 STROBE checklist.

## Additional Information and Declarations

### Data Availability

The following information was supplied regarding data availability:

The data is available in the Supplemental Files.
