# Supplementary material for: Predicting non-surgical treatment failure in patients with spontaneous pneumothorax—the Base-C score: a retrospective study
Source: PeerJ. 2026 Jun 18;14:e21288. doi: 10.7717/peerj.21288 (PMC13283364; doi:10.7717/peerj.21288)
Supplement: Supplemental Information 1 [file peerj-14-21288-s001.docx]

Supplementary Table 1 Calibration of the Base-C Score Across Risk Deciles in the Derivation Cohort.

| **Base-C Score Decile** | **Score Range** | **N** | **Predicted risk (%)** | **Actual risk (%)** |
| --- | --- | --- | --- | --- |
| 1 | 0 | 51 | 3.68(3.55-3.81) | 11.76 |
| 2 | 0-1 | 51 | 5.14(4.25-6.02) | 3.92 |
| 3 | 1-1 | 51 | 10.64(10.26-11.01) | 11.76 |
| 4 | 1-2 | 51 | 15.88(14.78-16.97) | 17.65 |
| 5 | 2-2.5 | 51 | 19.43(17.69-21.16) | 15.69 |
| 6 | 2.5-3 | 51 | 37.26(35.29-39.24) | 15.67 |
| 7 | 3-4.5 | 50 | 53.07(51.04-55.11) | 50.00 |
| 8 | 4.5-5.5 | 50 | 78.93(76.29-81.58) | 80.00 |
| 9 | 5.5-6.5 | 50 | 89.87(89.01-90.72) | 90.00 |
| 10 | 6.5-8 | 50 | 96.06(95.49-96.62) | 94.00 |
