# Supplementary material for: Predicting non-surgical treatment failure in patients with spontaneous pneumothorax—the Base-C score: a retrospective study
Source: PeerJ. 2026 Jun 18;14:e21288. doi: 10.7717/peerj.21288 (PMC13283364; doi:10.7717/peerj.21288)
Supplement: Supplemental Information 2 [file peerj-14-21288-s002.docx]

Supplementary Table 2: Predictive Performance of the Base-C Score at the Optimal Cut-off Value (≥4 points) Determined by Youden’s Index

| **Cohort** | **Cut-off** | **Sensitivity (%)** | **Specificity (%)** | **Youden‘s Index (J)** |
| --- | --- | --- | --- | --- |
| **Derivation**​ (n=1378) | 4 | 0.713 | 0.869 | 0.582 |
| **Validation**​ (n=506) | 4 | 0.638 | 0.855 | 0.493 |
