# Supplementary figures and images for: Predicting non-surgical treatment failure in patients with spontaneous pneumothorax—the Base-C score: a retrospective study
Source: PeerJ. 2026 Jun 18;14:e21288. doi: 10.7717/peerj.21288 (PMC13283364; doi:10.7717/peerj.21288)

**A****PSP**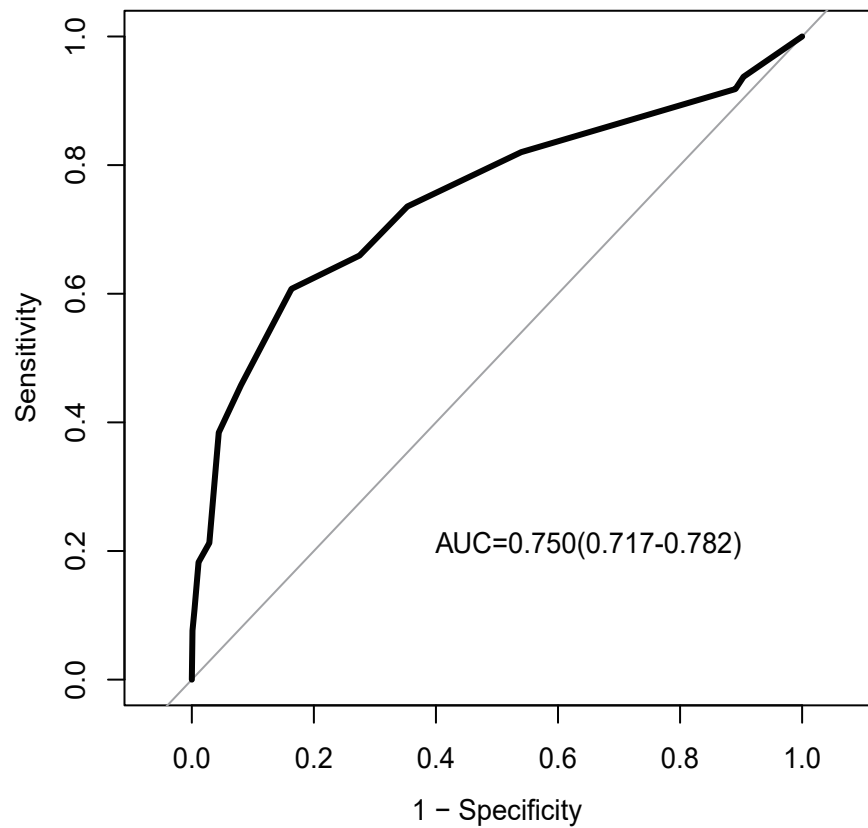**B****SSP**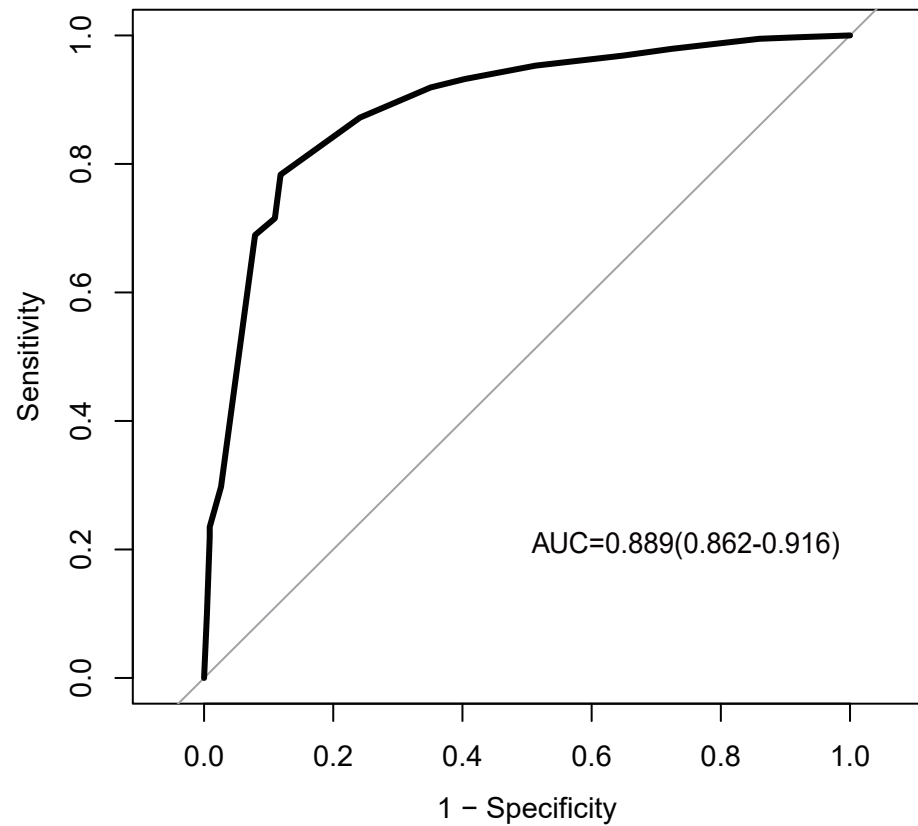

Supplement: Supplemental Information 3 [file peerj-14-21288-s003.pdf]

**A****PSP**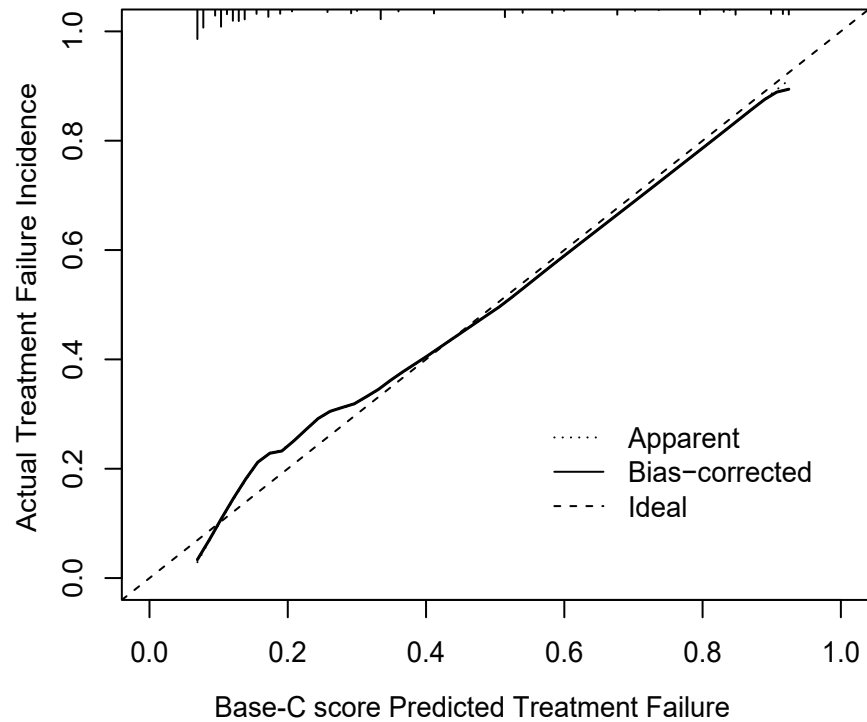**B****SSP**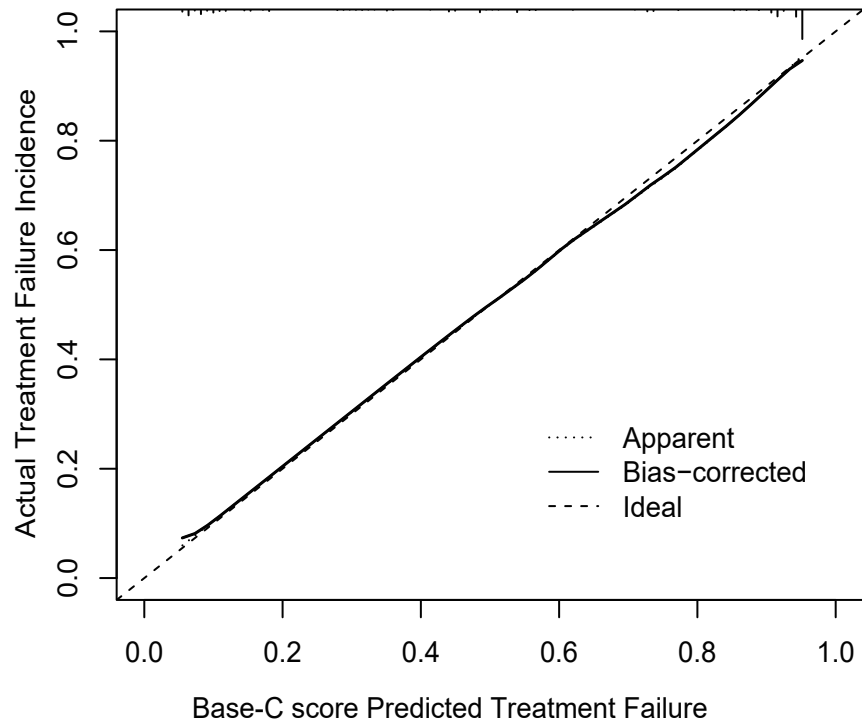

Supplement: Supplemental Information 4 [file peerj-14-21288-s004.pdf]

**A****PSP**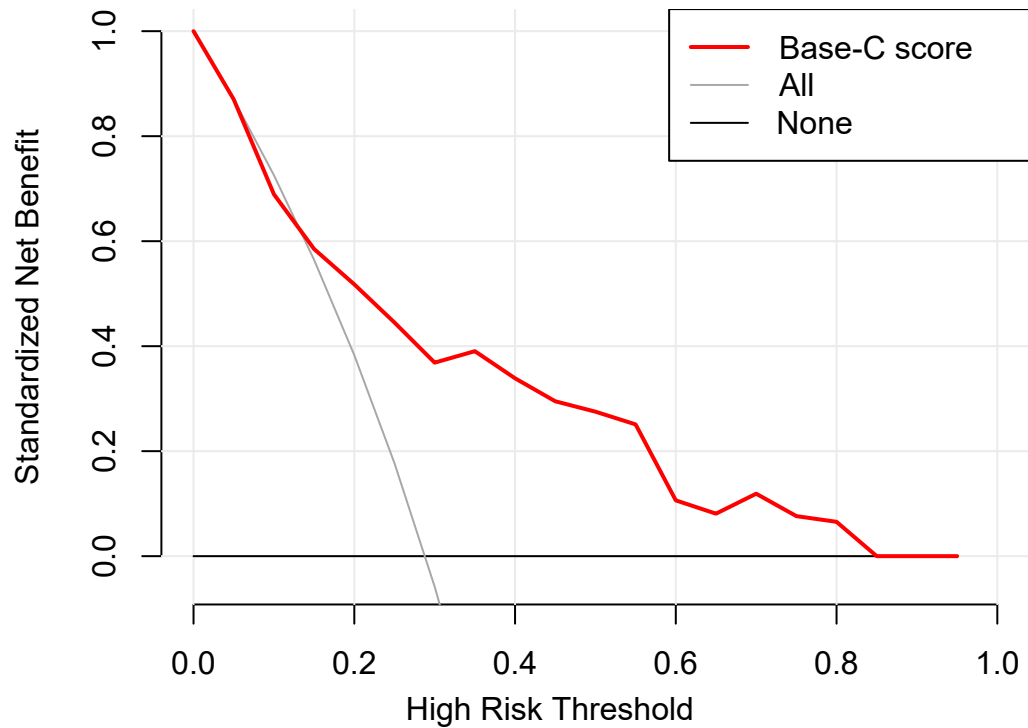**B****SSP**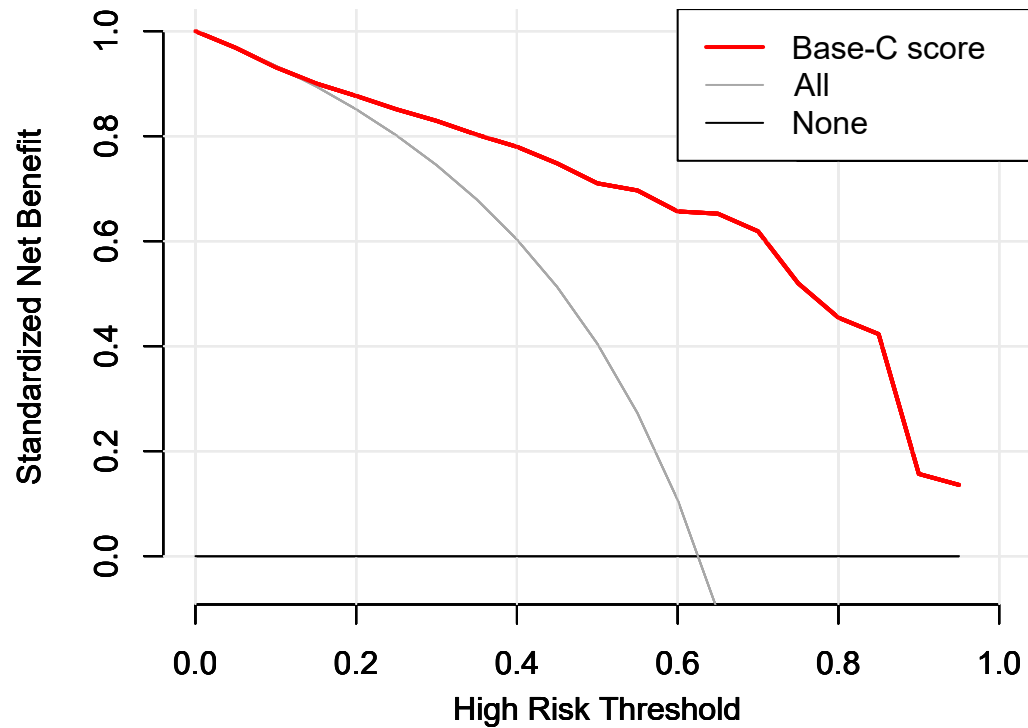

Supplement: Supplemental Information 5 [file peerj-14-21288-s005.pdf]
